# Molecular Cloning, Structural Analysis and Tissue Expression of *Protein Phosphatase 3 Catalytic Subunit Alpha Isoform* (*PPP3CA*) Gene in Tianfu Goat Muscle

**DOI:** 10.3390/ijms15022346

**Published:** 2014-02-07

**Authors:** Lu Wan, Jisi Ma, Gangyi Xu, Daihua Wang, Nianlu Wang

**Affiliations:** 1 Institute of Animal Genetics and Breeding, College of Animal Science and Technology, Sichuan Agricultural University, Ya’an 625014, Sichuan, China; E-Mails: wanlu19880605@163.com (L.W.); roy_0820@163.com (J.M.); luckymile@126.com (N.W.); 2 Mianyang Agriculture Bureau, Mianyang 621000, Sichuan, China; E-Mail: 13890178877@163.com

**Keywords:** *PPP3CA*, cDNA clone, sequence analysis, structure prediction, expression analysis

## Abstract

Calcineurin, a Ca^2+^/calmodulin-dependent protein phosphatase, plays a critical role in controlling skeletal muscle fiber type. However, little information is available concerning the expression of calcineurin in goat. Therefore, *protein phosphatase 3 catalytic subunit alpha isoform* (*PPP3CA*) gene, also called *calcineurin Aα*, was cloned and its expression characterized in Tianfu goat muscle. Real time quantitative polymerase chain reaction (RT-qPCR) analyses revealed that Tianfu goat *PPP3CA* was detected in cardiac muscle, biceps femoris muscle, abdominal muscle, longissimus dors muscle, and soleus muscle. High expression levels were found in biceps femoris muscle, longissimus muscle and abdominal muscle (*p* < 0.01), and low expression levels were seen in cardiac muscle and soleus muscle (*p* > 0.05). In addition, the spatial-temporal mRNA expression levels showed different variation trends in different muscles with the age of the goats. Western blotting further revealed that PPP3CA protein was expressed in the above-mentioned tissues, with the highest level in biceps femoris muscle, and the lowest level in soleus muscle. In this study, we isolated the full-length coding sequence of Tianfu goat *PPP3CA* gene, analyzed its structure, and investigated its expression in different muscle tissues from different age stages. These results provide a foundation for understanding the function of the *PPP3CA* gene in goats.

## 1. Introduction

Muscle fiber, significantly affecting the meat quality of livestock, has become a focus research area [1–3]. There are four muscle fiber types, including, slow-oxidative (Type I) muscle fibers, fast oxido-glycolytic (Type IIA) muscle fibers, and fast glycolytic (Type IIX and IIB) muscle fibers [4,5]. One study found that the main way to improve livestock meat quality is meant to raise the proportion of slow-oxidative (Type I) muscle fibers [6].

Calcineurin (CaN), also named protein phosphatase 3 (formerly 2B), is the only serine/threonine protein phosphatase under the control of Ca^2+^/calmodulin, and plays a critical role in the coupling of Ca^2+^ signals to cellular responses [7]. Ca^2+^ signaling plays a central role in hypertrophic growth of cardiac and skeletal muscle in response to mechanical load and a variety of signals [8]. Therefore, calcineurin plays an important role in muscle differentiation, especially in muscle fiber type conversion. It can differentially regulate genes in oxidative muscle fiber type fast myosin heavy chain conversion, evidently affecting the livestock meat quality [9–13]. On further study of the effects of calcineurin on muscle differentiation, it seems that calcineurin regulates muscle fiber types of muscle growth, depending both on muscle phenotype and stage of myofiber growth, using the calcineurin inhibitor cyclosporin A (CsA) [14,15]. This illustrates that *calcineurin*, is regarded as a candidate gene, that can play a role in muscle fiber differentiation, affecting the meat quality of livestock [16].

Regardless of the source of calcineurin, calcineurin is always a heterodimer of a 58–64 kD catalytic and calmodulin-binding subunit, calcineurin A (CnA), tightly bound to a regulatory 19 kD Ca^2+^-binding regulatory subunit, calcineurin B (CnB) [17]. CnA can be coded by three different genes, respectively, *PPP3CA* (isoform α), *PPP3CB* (isoform β) and *PPP3CC* (isoform γ), while CnB is encoded by two different genes, respectively, *PPP3R1* (CnB1) and *PPP3R2* (CnB2) [18]. Of all these genes, *PPP3CA* gene is highly expressed in skeletal muscle and takes a part in slow muscle fiber type switching [19]. *PPP3CA* gene, is widely distributed [7,20–23], has also been cloned in human, mouse, pig and cattle, and its encoded-protein is highly conservative [24–27].

*PPP3CA* gene has been isolated and its expression analyzed in other mammals already, but there is little to report on caprine. In our study, we cloned Tianfu goat *PPP3CA* gene, analyzed its cDNA sequence and encored-protein sequence, and examined its expression in different muscle tissues by real time quantitative polymerase chain reaction (RT-qPCR) and western blotting. The results of this study provide a foundation for understanding the function of the *PPP3CA* gene in muscle fiber type conversion.

## 2. Results and Discussion

### 2.1. Summary of Characteristics of Tianfu Goat *PPP3CA* Sequences and Structures

**Figure 1.**
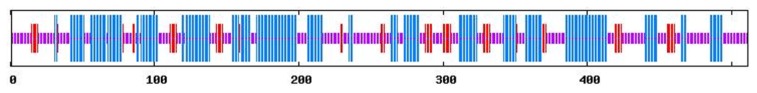
Predicted secondary structure of Tianfu goat PPP3CA amino acid (AA) sequence. The blue line represents α-helix, the red line represents extended strand, and the purple line represents random coil.

The cDNA nucleotide sequence analysis revealed that the nucleotide sequence represented one open reading frame (ORF), which contained 1536 bp encoded 511 amino acids (Supplementary Information). A BLAST search of NCBI’s nucleotide sequence database revealed that the fragment was significantly similar to the *PPP3CA* gene sequences from other mammals. The deduced amino acid sequence of Tianfu goat *PPP3CA* gene, having a molecular weight of 58.0 kDa and pI (isoelectric point) of 5.91, was analyzed with the ProtParam tool. The Tianfu goat PPP3CA protein was not a signal peptide, and had no obvious transmembrane domain. There were 33 phosphorylation sites successfully predicted by the neural network in Tianfu goat PPP3CA protein. The secondary structure of the PPP3CA protein was predicted to be mainly α-helix (43.25%) and random coil (45.79%) (Figure 1). A conserver domain, named PP2Ac, was calculated using the NCBI CD-search and the SMART. The PP2Ac domain, from 56 to 347 AA, was a part of protein phosphatase 2A homologues catalytic domain, a large family of serine phosphatases, which includes PP1, PP2A and PP2B (calcineurin) family members, inferring the conserved domain of PP2Ac, having a similar function with serine phosphatases, and maybe a functional area.

**Figure 2.**
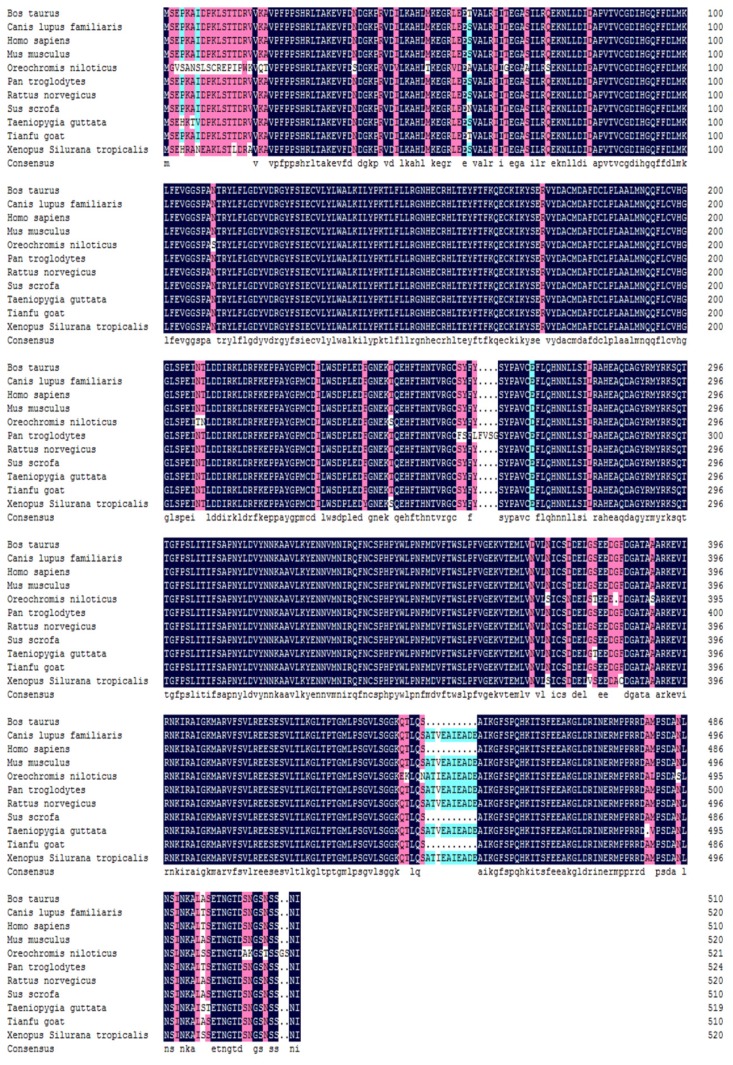
Amino acid sequence alignment of PPP3CA from different species. The blue, pink and blue-green represent the different levels of amino acid sequence similarity of PPP3CA from different species (high, intermediate, and low similarity, respectively).

Sequence alignment using DNAMAN V6 software revealed that the coding nucleotide sequence of the Tianfu goat *PPP3CA* gene was 97.1% identical to bovine *PPP3CA*, and 96.7%, 93.9%, 91.2%, 90.6%, 95.6%, 92.7%, 86.8%, 83.2% and 76.2% identical to porcine, dog, mice, rat, human, chimpanzee, finch, frog and tilapia, respectively, and the amino acid sequences were 99.8%, 99.6%, 97.7%, 97.7%, 97.7%, 99.6%, 96.0%, 96.2%, 95.0% and 87.2% identical to bovine, porcine, dog, mice, rat, human, chimpanzee, finch, frog and tilapia, respectively (Figure 2). Sequence alignment revealed that PPP3CA proteins from different species were highly conservative.

**Figure 3.**
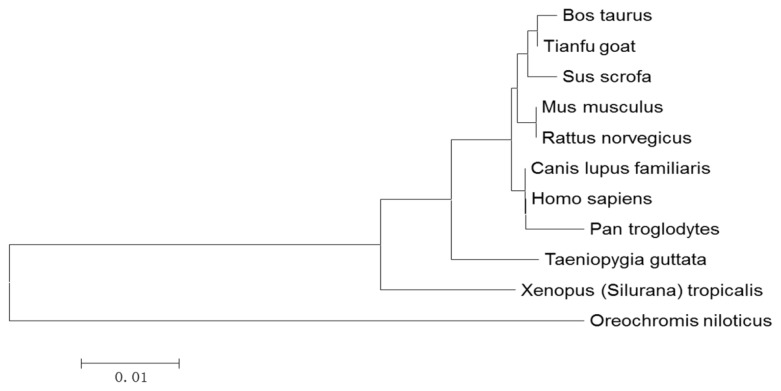
Phylogenetic tree of the amino acid sequences of PPP3CA. The tree was constructed using the Neighbor Joining method in the MEGA 5.10 software. All above sequences are from the NCBI data base (*Bos mutus*: NP_777212.1; *Sus scrofa*: NP_999293.1; *Homo sapiens*: NP_000935.1; *Pan troglodytes*: XP_001168163.1; *Canis lupus familiaris*: NP_001184025.1; *Mus musculus*: NP_058737.1; *Rattus norvegucus*: NP_058737.1; *Taeniopygia guttate*: XP_002193146.2; *Oreochromis niloticus*: XP_004554421.1; and *Xenopus (Silurana) tropicalis*: NP_001119980.1).

Amino acid sequences of PPP3CA from 11 species were used for the phylogenetic tree constructed with the software MEGA5.10. The results showed that all of the PPP3CA proteins from different animals were divided into one subgroup. The highest homology was with cattle, the lowest homology was with tilapia (bony fishes) (Figure 3).

**Figure 4.**
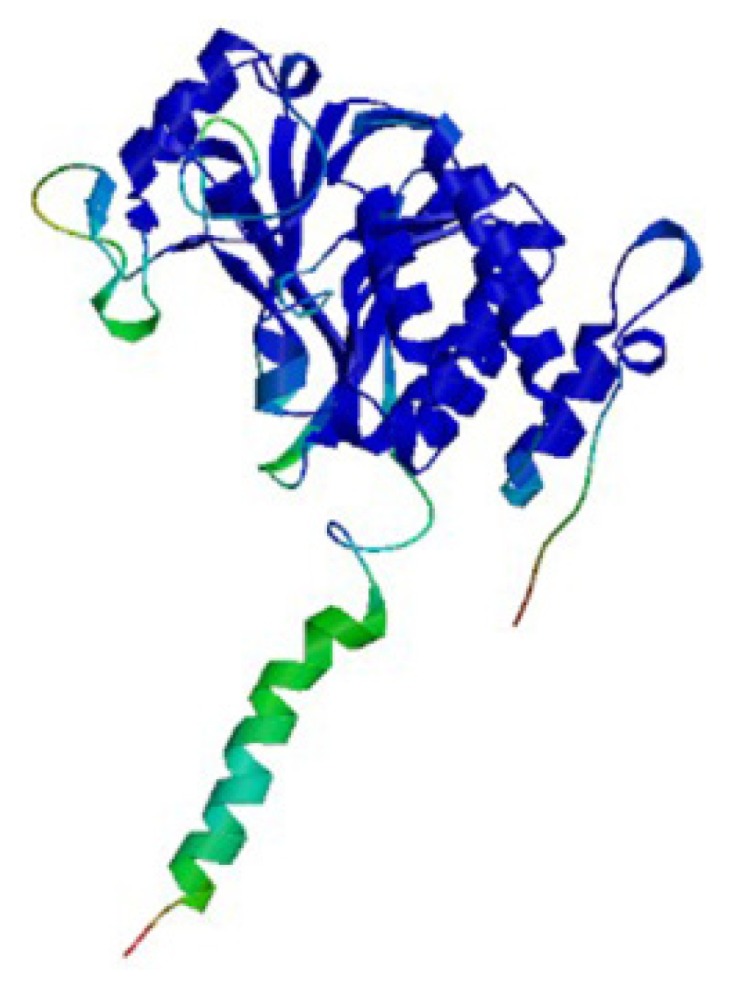
The 3D structural model of Tianfu goat PPP3CA (1–352 AA) based on homology modeling.

**Figure 5.**
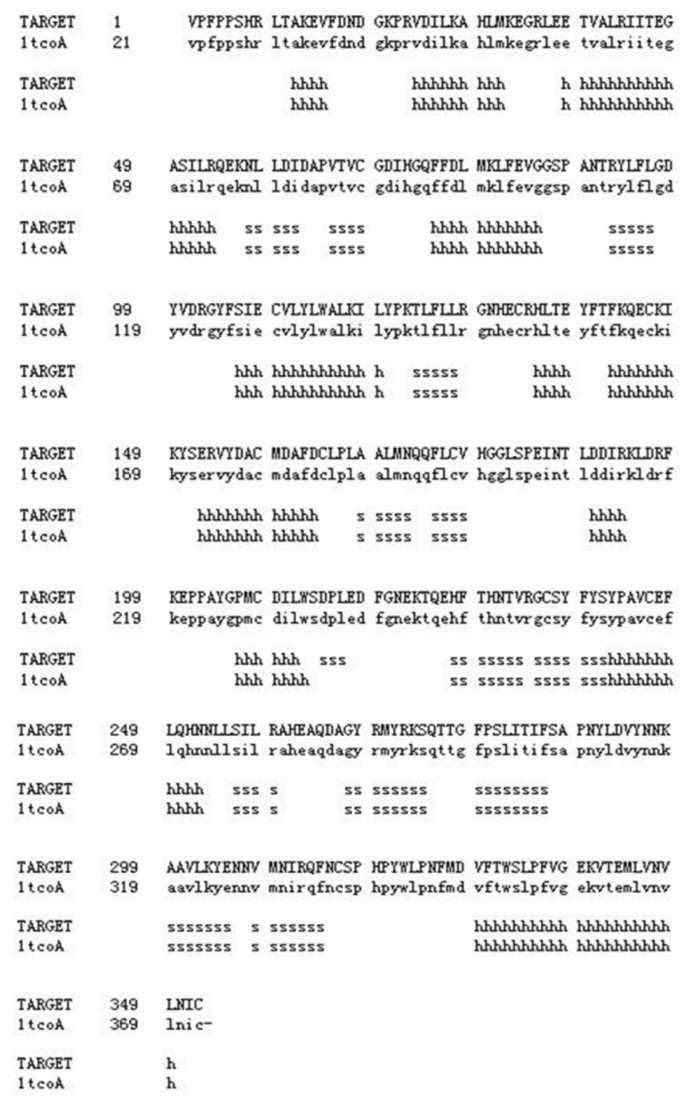
Alignment of the Tianfu goat PPP3CA and ZN505 amino acid sequences. TARGET represents Tianfu goat PPP3CA (1–352 AA), 1tcoA represents ZN505 (21–372 AA); s indicates the residues that form the “bend”; and h indicates the residues that form the “α-helix”.

A SWISS-MODEL server was used to construct a 3D structural model of Tianfu goat PPP3CA amino acid sequence (between amino acids (AA) 1–352). The homology modeling revealed that this segment was similar to that of the ZN505 in the Protein Data Bank (ZN505: 1tcoA: 21–372 AA) (Figure 4). In Figure 5, there is no difference between PPP3CA (1–352 AA) protein domain and ZN505-1tcoA (21–372 AA) domain. The 3D structural model of Tianfu goat may provide a basis for further studying the relationship between structure and function of PPP3CA protein in goats.

### 2.2. Analysis of mRNA Expression of Tianfu Goat *PPP3CA* in Different Muscle

**Figure 6.**
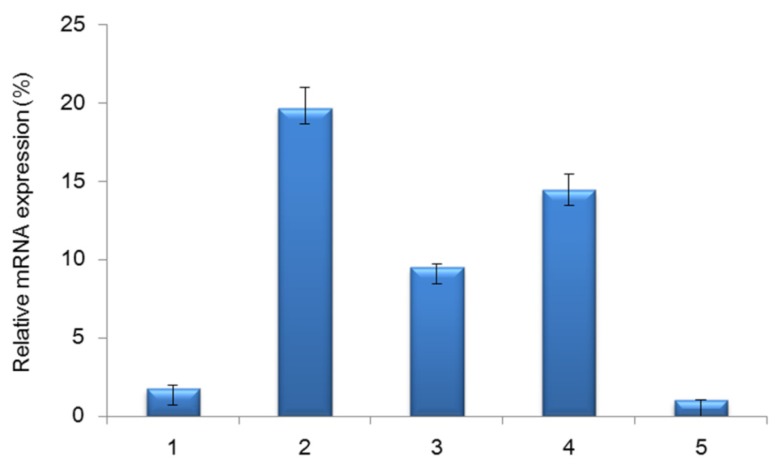
Spatial mRNA expression profile of Tianfu goat *PPP3CA* gene. The samples 1–5 represent cardiac muscle, biceps femoris muscle, abdominal muscle, longissimus dors muscle and soleus muscle, respectively. Bars represent the mean ± SD (*n* = 8).

RT-qPCR was conducted to determine the spatial-temporal mRNA expression of *PPP3CA* gene in Tianfu goat muscle tissues, employing glyceraldeyhyde-3-phosphate dehydrogenase (GAPDH) as endogenous control in each sample. The RT-qPCR results showed that Tianfu goat *PPP3CA* mRNA existed in cardiac muscle, biceps femoris muscle, abdominal muscle, longissimus dors muscle and soleus muscle at 150 days old (Figure 6). Particularly, high expression levels of *PPP3CA* were found in biceps femoris muscle, longissimus dors muscle and abdominal muscle (*p* < 0.01), and low expression levels were observed in cardiac muscle and soleus muscle (*p* > 0.05). This result is consistent with previous research in pig (Figure 6) [26]. In former studies, Pette *et al*., showed that the muscle fiber type changed after birth [28]. That is to say, proportions of fast and slow muscle fiber types from different muscle tissues were different. It stands to reason that the *PPP3CA* gene was differently expressed in different muscle tissues with different proportions of fast and slow muscle fibers, suggesting the *PPP3CA* gene is the key to muscle fiber growth and development [22,26].

**Figure 7.**
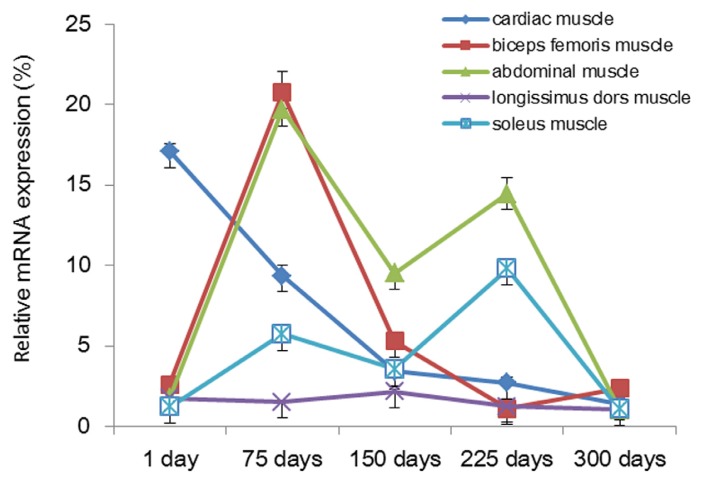
Temporal mRNA expression profiles of *PPP3CA* during different muscle development in Tianfu goat. Bars represent the mean ± SD (*n* = 8).

In addition, the *PPP3CA* spatial-temporal mRNA expression levels in different muscles showed different variation trends in five muscle tissues with the age of Tianfu goats. During cardiac muscle development, gene expression of *PPP3CA* was decreased gradually from the day 1 to the day 300; the highest expression level was seen on the day 1 and the lowest expression level on the day 300 (Figure 7). In biceps femoris muscle, gene expression of *PPP3CA* was first increased from the day 1 to the day 75, then decreased to the day 225 and finally increased to the day 300; the highest expression was seen on the day 75 and the lowest on the day 225 (Figure 7). In abdominal muscle, gene expression of *PPP3CA* was first increased from the day 1 to the day 75, decreased to the day 150, after which it increased to the day 225, and finally decreased to the day 300; the highest expression was observed on the day 75 and the lowest on the day 300 (Figure 7). This contrasts with *PPP3CA* gene expression in soleus muscle, which was highest on the day 225 and lowest on the day 300. In longissimus dors muscle, gene expression of *PPP3CA* was first decreased from the day 1 to the day 75, then increased to the day 150 and finally decreased to the day 300; the highest expression was seen on the day 150 and the lowest on the day 300 (Figure 7). Among these trends, the change curves of gene expression of *PPP3CA* appeared to have remarkable fluctuation over the period from the day 1 to the day 300 in cardiac muscle, biceps femoris muscle, abdominal muscle, and soleus muscle (Figure 7). However, during that time, there was a slight change of *PPP3CA* gene expression in longissimus dors muscle (Figure 7).

McCoard *et al.*, previously found that the proportions of slow muscle fibers grew steadily in different muscles of lamb [29]. During muscle growth and development, the mRNA expression of *PPP3CA* gene showed different variation trends in five muscle tissues with different proportions of slow muscle fiber (Figure 7), implying *PPP3CA* could take a major part in slow muscle fiber differentiation [23]. However, a special trend, a slight variation trend of *PPP3CA* gene expression, was found in longissimus dors muscle (Figure 7). After birth, muscle fibers of longissimus dors muscle were found primarily to have more fast muscle fibers than their counterparts in other muscle tissues, signifying they were differentiated into fast muscle fibers in longissimus dors muscle [1,30]. Consequently, gene expression of *PPP3CA* slowed varying transformation of fast muscle fibers in longissimus dors muscle from different age stages of Tianfu goats. These results further confirmed that *PPP3CA* may serve a function in controlling slow muscle fiber differentiation [19].

### 2.3. Western Blotting Analysis of Protein Expression of Tianfu Goat PPP3CA

**Figure 8.**
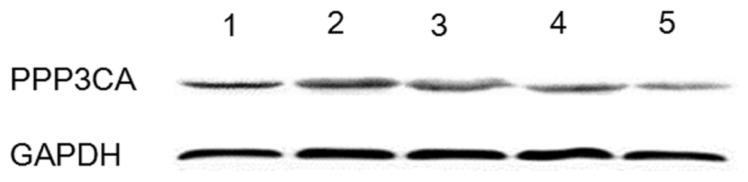
Western blotting of Tianfu goat PPP3CA protein levels in five muscle tissues. The samples 1–5 represent cardiac muscle, biceps femoris muscle, abdominal muscle, longissimus dors muscle and soleus muscle, respectively, (*n* = 8).

Western blotting results showed different expression levels of PPP3CA protein in cardiac muscle, biceps femoris muscle, abdominal muscle, longissimus dors muscle, and soleus muscle at 150 days old. Using GAPDH as the reference protein in each sample, PPP3CA protein was expressed in the above-mentioned muscle tissues, with the highest level in biceps femoris muscle and the lowest level in soleus muscle (Figure 8).

During the muscle fiber development and differentiation, the slow muscle fibers grew steadily in lamb muscles, but with the exception of soleus muscle [29,31]. This means that the slow muscle fiber proportions of soleus muscle were not significantly changed after birth [31]. *PPP3CA* came into play a role in slow muscle fiber differentiation, the more the gene expression of *PPP3CA*, the stronger its effects in slow muscle fiber differentiation [9,10,19]. Thereby, PPP3CA protein was expressed at the lowest level in soleus muscle [19]. The results of western blotting further confirmed that PPP3CA protein was differently expressed in different muscle tissues with different proportions of muscle fibers. This threatens *PPP3CA* gene required for the key processes of myocyte differentiation and conversion to the slow muscle fiber type [7,12,19].

## 3. Experimental Section

### 3.1. Animals and Sample Collection

All experimental procedures were approved by Sichuan Agricultural University Animal Care and Use Committee, Sichuan Agricultural University, Sichuan, China under permit No. DKY-B20100805. All of the Tianfu goats were bred under the same standard conditions and slaughtered on the 1st, 75th, 150th, 225th, and 300th day after birth (*n* = 8 per group). The five muscle samples, including cardiac muscle, biceps femoris muscle, abdominal muscle, longissimus dors muscle and soleus muscle, were harvested, frozen in liquid nitrogen, and stored at −80 °C until total RNA and protein extraction.

### 3.2. Total RNA Isolation and Synthesis of cDNA

According to the manufacturer protocol’s, total RNA was extracted from five muscle tissues (cardiac muscle, biceps femoris muscle, abdominal muscle, longissimus dors muscle, and soleus muscle) using the RNAiso Plus (TaKaRa, Dalian, China). Total RNA was detected by 1% agarose gel electrophoresis with 10× loading buffer (Tiangen, Beijing, China), and then preserved at −80 °C. According to the manufacturer’s protocol, the first strand cDNA was compounded using a PrimeScript™ RT Reagent kit (TaKaRa). First strand cDNA was obtained and preserved at −20 °C a freezer.

### 3.3. Cloning of the Tianfu Goat *PPP3CA* Gene

To clone the cDNA sequence of Tianfu goat *PPP3CA* gene, the RNAs were extracted from the longissimus dorsi muscle of Tianfu goat with the following cDNA library construction. A pair of primers, 5′-ATGTCCGAGCCCAAGGCAATT-3′ and 5′-AGCAGCAATATCCAGTGA-3′, which was designed by the Primer premier 5.0 software (PREMIER Biosoft International, Palo Alto, Canada) and based on the bovine *PPP3CA* gene (GenBank No. NM_174787.2) [32] was prepared for PCR. PCRs were run under the following cycling conditions: 95 °C for 5 min, followed by 40 cycles of 95 °C for 30 s; 61.4 °C for 30 s; 72 °C for 90 s, and a final extension at 72 °C for 10 min. The products of PCRs were detected by 1.5% agarose gel electrophoresis, and then the PCR products were obtained. An E.Z.N.A Gel Extraction Kit (Omega BioTek, Doraville, GA, USA) was used to extract the PCR fragments from the gel, and then ligated into pMD19-T vector (TaKaRa) at 4 °C overnight. Finally, the cloned products were sequenced by LiuHe HuaDa Biotechnology Co. Ltd. (Beijing, China).

### 3.4. Sequence Analysis

Sequence analysis of the predicted PPP3CA protein of Tianfu goat translated used ExPaSy (http://www.expasy.org). The molecular weight and pI (isoelectric point) were divined by ProtParam tool (http://web.expasy.org/protparam/). The NetPhos 2.0 server (http://www.cbs.dtu.dk/services/NetPhos/) was used to produce neural network predictions for phosphorylation sites in Tianfu goat PPP3CA protein, while SignalP 4.1 server (http://www.cbs.dtu.dk/services/SignalP/) was employed to dope out location of signal peptide cleavage sites in PPP3CA amino acid sequence. The transmembrane helices were calculated to preforecast by TMHMM 2.0 (http://www.cbs.dtu.dk/services/TMHMM-2.0/). The putative conserved domain of PPP3CA protein was calculated by NCBI Batch Web CD-search tool (http://www.ncbi.nlm.nih.gov/Structure/bwrpsb/bwrpsb.cgi) and SMART (http://smart.embl-heidelberg.de/smart/set_mode.cgi?NORMAL=1) online tool. The secondary structure of deduced amino acid sequence was predicted by NPSA (http://npsa-pbil.ibcp.fr/cgi-bin/npsa_automat.pl?page=/NPSA/%20npsa_hnn.html\). The coding nucleotide and protein sequences of PPP3CA from related different species were aligned with the Tianfu goat sequences using the DNAMAN V6 software (Lynnon Biosoft, Los Angeles, CA, USA). Amino acid sequences of PPP3CA protein from 11 species were used for the phylogenetic tree constructed by the MEGA5.10 program. The SWISS-MODEL server (http://www.expasy.org/swissmod/SWISSMODEL.html) was used to model the Tianfu goat PPP3CA protein 3D conformation.

### 3.5. RT-qPCR Analysis of *PPP3CA* Gene mRNA Expression

Total RNAs of five muscle tissues were extracted. RT-qPCR was used to analyze the relative mRNA expression of *PPP3CA* gene in different muscle tissues of Tianfu goat at 150 days old. Analysis of spatial-temporal expression patterns of *PPP3CA* gene used the housekeeping gene, *GAPDH* gene as an internal control. The primers are shown in Table 1. The 25 μL reaction system of RT-qPCR was: 12.5 μL SYBR premix Ex TAq™ (TaKaRa), 1 μL Forward primer (4.19 nmol per OD), 1 μL Reverse primer (4.37 nmol per OD), 2 μL cDNA of each muscle tissue, 8.5 μL RNase-free H_2_O (Tiangen). RT-qPCR program initially started with: 95 °C for 10 s followed by 40 cycles of 95 °C for 5 s and 60 °C for 30 s.

### 3.6. Western Blotting Analyses of PPP3CA Protein Expression

Total proteins of five muscle tissues were extracted by using a Total Protein Extraction Kit (Sangon Biotech, Shanghai, China), according to the manufacturer’s protocol, and then the muscle proteins were preserved at −80 °C in a freezer. Western blotting was used to analyze the relative protein expression of PPP3CA in muscle tissues of Tianfu goat at 150 days old. The determination of the protein concentration used BCA protein assay kit (Beyotime, Shanghai, China) with bovine serum albumin (BSA) as a standard. GAPDH protein was selected as an internal control. Polyclonal antibodies to the calcineurin protein (Abcam, Cambridge, UK) were used at 1/500 dilution and a monoclonal antibody to the GAPDH protein (BiYunTian, Shanghai, China) was at a 1/1000 dilution. Equal amounts of protein (12 μg per lane) were resolved on 12% SDS-polyacrylamide gels, and then as detailed previously in [33], the intensity of the signal was used to analyze the relative amount of PPP3CA protein in Tianfu goat muscle tissues extracts.

### 3.7. Statistical Analysis

All data are expressed as the means ± SD. Statistical analysis was performed using one-way analysis of variance (ANOVA) followed by Duncan’s multiple-range test through the SPSS 13.0 statistical software package (SPSS Inc., Chicago, IL, USA). The threshold of significance was defined as *p* < 0.05.

## 4. Conclusions

In our study, we isolated the Tianfu goat *PPP3CA* gene and used various bioinformatics software to analyze its nucleotide and protein sequences. We predicted a 3D structure of PPP3CA protein (1–352 AA) and analyzed the differences of spatial-temporal mRNA expression in different muscle tissues from five age stages of Tianfu goats. Additionally, western blotting was used to analyze the expression of PPP3CA protein in five muscle tissues of Tianfu goats. The first evidence showed that *PPP3CA* gene is expressed in goat muscle tissues and has an expression pattern similar to porcine, suggesting that a similar function of *PPP3CA* in both goats and pigs. This information obtained provides an important theoretical basis for further research into the function of *PPP3CA*.

## Supplementary Information



## Figures and Tables

**Table 1.** Primer pairs used for analysis of the gene expression of Tianfu goat *PPP3CA*.

Primer	Sequence fragment	Product length (bp)	Application
PPP3CA-F	GAACCACCTGCTTATGGACCTAT	244	Expression
PPP3CA-R	AAGGGAAGCCTGTTGTTTGG	244	Expression
GAPDH-F	GTCACCAACTGGGACGACA	118	RT-qPCR
GAPDH-R	AGGCGTACAGGGACAGCA	118	RT-qPCR
