# Supplementary material for: Molecular Cloning, Structural Analysis and Tissue Expression of Protein Phosphatase 3 Catalytic Subunit Alpha Isoform (PPP3CA) Gene in Tianfu Goat Muscle
Source: Int J Mol Sci. 2014 Feb 7;15(2):2346–58. doi: 10.3390/ijms15022346 (PMC3958854; doi:10.3390/ijms15022346)
Supplement: Supplementary file 1 [file ijms-15-02346-s001.pdf]

Supplementary Information

Tianfu goat-nucleotide sequence

ATGTCCGAGCCCAAGGCAATTGATCCCAAGTTGTCGACGACCGACAGGGTGGTGAAAGCT
GTCCCATTTCCTCCAAGTCACCGGCTTACGGCAAAAGAAGTGTTTGATAACGATGGGAAAC
CTCGTGTGGATATCTTAAAGGCACATCTCATGAAGGAGGGCAGGCTCGAAGAGACTGTCGC
ATTGAGAATAATAACAGAGGGGGCTTCAATTCTTCGACAGGAAAAAAACTTGCTGGATATT
GATGCTCCAGTCACAGTTTGTGGGGACATCCATGGACAATTCTTTGACTTGATGAAACTCTT
TGAAGTGGGGGGATCTCCTGCCAACACTCGCTACCTCTTCTTAGGGGACTATGTTGACAGA
GGGTACTTCAGTATCGAATGTGTGCTGTATTTGTGGGCTTTGAAAATTCTTTACCCCAAAAC
ACTGTTTTTACTTCGTGGAAATCATGAATGTAGACATCTAACAGAGTATTTCACATTTAAAC
AAGAATGTAAAATAAAGTATTCAGAACGTGTATATGATGCCTGCATGGATGCCTTTGACTGC
CTGCCCCTGGCTGCCCTGATGAACCAGCAGTTCCTGTGTGTACACGGCGGTTTGTCTCCAG
AGATAAACACTTTAGATGATATCAGAAAATTAGACCGATTCAAAGAACCACCTGCTTATGGA
CCTATGTGTGATATCCTGTGGTCAGACCCGCTGGAAGATTTTGGAAATGAGAAGACTCAGG
AACATTTCACTCACAACACAGTCAGGGGCTGTTCATACTTCTACAGTTACCCGGCTGTATGT
GAATTCTTGCAGCACAATAACTTGTTATCTATCCTCCGAGCTCACGAAGCCCAAGATGCAG
GGTACCGCATGTACAGGAAAAGCCAAACAACAGGCTTCCCTTCTCTCATTACAATTTTTC
AGCACCAAATTACTTAGATGTATACAATAACAAAGCTGCAGTATTGAAGTATGAGAACAATG
TCATGAATATCAGGCAATTCAACTGTTCTCCTCATCCATACTGGCTTCCAAATTTCATGGATG
TTTTCACCTGGTCCCTGCCATTTGTTGGGGAAAAAGTGACTGAGATGCTGGTAAATGTCCT
CAACATCTGCTCAGACGATGAACTGGGGTCAGAAGAAGATGGATTTGATGGAGCAACGGC
TGCGGCCAGGAAGGAGGTGATAAGAAATAAGATCCGAGCCATCGGCAAGATGGCCAGAGT
GTTCTCGGTTCTCAGAGAAGAGAGTGAAAGTGTGCTGACGCTGAAAGGCCTGACGCCCAC
AGGCATGCTCCCCAGCGGCGTGCTTTCTGGAGGGAAACAAACCCTGCAAAGCGCTATCAA
AGGATTTTCACCACAACATAAGATCACTAGCTTCGAGGAGGCCAAGGGCTTAGACCGAATT
AACGAGAGGATGCCGCCTCGCAGAGATGCCATGCCCTCTGACGCCAACCTTAACTCCATCA
ACAAGGCTCTCGCCTCAGAGACTAACGGCACGGACAGCAATGGCAGTAATAGCAGCAATA
TCCAGTGA

Tianfu goat-amino acid sequence

MSEPKAIDPKLSTTDRVVKAVPFPPSHRLTAKEVFDNDGKPRVDILKAHLMKEGRLEETV
ALRIITEGASILRQEKNLLDIDAPVTVCGDIHGQFFDLMKLFEVGGSPANTRYLFLGDYV
DRGYFSIECVLYLWALKILYPKTLFLLRGNHECRHLTEYFTFKQECKIKYSERVYDACMD
AFDCLPLAALMNQQFLCVHGGLSPEINTLDDIRKLDRFKEPPAYGPMCDILWSDPLEDFG
NEKTQEHFTHNTVRGCSYFYSYPAVCEFLQHNNLLSILRAHEAQDAGYRMYRKSQTTGFP
SLITIFSAPNYLDVYNNKAAVLKYENNVMNIRQFNCSPHPYWLPNFMDVFTWSLPFVGEK
VTEMLVNVLNICSDDELGSEEDGFDGATAAARKEVIRNKIRAIGKMARVFSVLREESESV
LTLKGLTPTGMLPSGVLSGGKQTLQSAIKGFSPQHKITSFEEAKGLDRINERMPPRRDAM
PSDANLNSINKALASETNGTDSNGSNSSNIQ
